# Hematopoietic Disorders, Renal Impairment and Growth in Mucopolysaccharidosis-Plus Syndrome

**DOI:** 10.3390/ijms23105851

**Published:** 2022-05-23

**Authors:** Viktoriia Sofronova, Rina Iwata, Takuya Moriya, Kiunniai Loskutova, Elizaveta Gurinova, Mairanush Chernova, Anastasia Timofeeva, Anna Shvedova, Filipp Vasilev, Saina Novgorodova, Seigo Terawaki, Takahito Moriwaki, Aitalina Sukhomyasova, Nadezhda Maksimova, Takanobu Otomo

**Affiliations:** 1 Department of Molecular and Genetic Medicine, Kawasaki Medical School, Kurashiki 701-0192, Japan; viksofmax92@gmail.com (V.S.); rina.iwata.0602@gmail.com (R.I.); terawaki@med.kawasaki-m.ac.jp (S.T.); moriwaki@med.kawasaki-m.ac.jp (T.M.); 2 Laboratory of Molecular Medicine and Human Genetics, North-Eastern Federal University, 677013 Yakutsk, Russia; vasilyevmd@gmail.com (F.V.); vsaina@yandex.ru (S.N.); aitalinas@yandex.ru (A.S.); nogan@yandex.ru (N.M.); 3 Department of Pathology, Kawasaki Medical School, Kurashiki 701-0192, Japan; tmoriya@med.kawasaki-m.ac.jp; 4 Department of Pathological Anatomy, Republic Hospital No. 1—National Center of Medicine, 677019 Yakutsk, Russia; loskutovaks@mail.ru; 5 Medical Institute, North-Eastern Federal University, 677013 Yakutsk, Russia; 6 Medical Genetics Center, Republic Hospital No. 1—National Center of Medicine, 677019 Yakutsk, Russia; elgur2005@yandex.ru; 7 Department of Children’s Health and Pathological Anatomy, Republic Hospital No. 1—National Center of Medicine, 677019 Yakutsk, Russia; timur31082011@mail.ru (M.C.); anastasia.1974.timofeeva@gmail.com (A.T.); metastasis228@gmail.com (A.S.)

**Keywords:** mucopolysaccharidosis, lysosomal storage disease, mucopolysaccharidosis-plus syndrome, VPS33A, proteinuria, renal failure, foamy podocyte, anemia, thrombocytopenia

## Abstract

Mucopolysaccharidoses (MPS) are rare lysosomal storage disorders (LSD) characterized by the excessive accumulation of glycosaminoglycans (GAG). Conventional MPS, caused by inborn deficiencies of lysosomal enzymes involved in GAG degradation, display various multisystemic symptoms—including progressive neurological complications, ophthalmological disorders, hearing loss, gastrointestinal and hepatobiliary issues, cardiorespiratory problems, bone and joint abnormalities, dwarfism, and coarse facial features. Mucopolysaccharidosis-Plus Syndrome (MPSPS), an autosomal recessive disease caused by a mutation in the endo-lysosomal tethering protein VPS33A, shows additional renal and hematopoietic abnormalities (“Plus symptoms”) uncommon in conventional MPS. Here, we analyze data from biochemical, histological, and physical examinations—particularly of blood counts and kidney function—to further characterize the clinical phenotype of MPSPS. A series of blood tests indicate hematopoietic symptoms including progressive anemia and thrombocytopenia, which correlate with histological observations of hypoplastic bone marrow. High urinary excretion of protein (caused by impairments in renal filtration), hypoalbuminemia, and elevated levels of creatinine, cholesterol, and uric acid indicate renal dysfunction. Histological analyses of MPSPS kidneys similarly suggest the extensive destruction of glomerular structures by foamy podocytes. Height and weight did not significantly deviate from the average, but in some cases, growth began to decline at around six months or one year of age.

## 1. Introduction

Lysosomal storage disorders (LSD) are a group of inborn errors of metabolism characterized by the accumulation of undegraded substances [1]. Inherited lysosomal enzyme deficiencies cause most LSDs, but the deficit of key proteins involved in autophagy, endocytosis, production/transportation of lysosomal enzymes, and other biological processes, may similarly lead to lysosomal dysfunction [2]. Mucopolysaccharidoses (MPS), a type of LSD, display excessive accumulation of glycosaminoglycans (GAG) including dermatan sulfate, heparan sulfate, keratan sulfate, chondroitin sulfate, and hyaluronan. Depending on the type of accumulated GAG, deficient enzyme, and the clinical phenotype, MPSs are categorized into seven groups: MPS I, MPS II, MPS III, MPS IV, MPS VI, MPS VII, and MPS IX [3,4]. All MPSs have an autosomal recessive inheritance pattern except for MPS II, which is X-linked. Common manifestations include progressive neurological complications and associated motor/cognitive decline, ophthalmological disorders, hearing loss, gastrointestinal and hepatobiliary issues, cardiorespiratory problems, bone and joint abnormalities, dwarfism, and coarse facial features—with varying levels of severity.

Mucopolysaccharidosis-Plus Syndrome (MPSPS) is a severe autosomal recessive disease caused by a mutation in the vacuolar-protein-sorting-associated protein 33A (*VPS33A*) gene. Patients exhibit an accumulation of heparan sulfate and dermatan sulfate, MPS-like manifestations, additional renal and hematopoietic symptoms absent in conventional MPS, and low life expectancy (10–20 months) [5]. Previously, we reported 13 cases in Yakutia, Russia [6,7]—and since then, two patients from a Turkish consanguineous family and one patient from a Moroccan consanguineous family have been reported [8,9]. Currently, a total of 18 cases are registered in Yakutia, and the specific p.R498W homozygous mutation in the *VPS33A* gene has been confirmed in all patients,. VPS33A is a key component in two tethering complexes—homotypic fusion and protein sorting (HOPS), and class C core vacuole/endosome tethering (CORVET)—which play a role in vesicle-mediated protein trafficking to lysosomes in autophagy and endocytosis [10]. However, MPSPS patient-derived skin fibroblasts do not show abnormal lysosomal enzyme activity, nor autophagy/endocytosis impairment [7]. Molecular analyses have revealed reduced levels of VPS33A, elevated concentrations of sphingolipid β-D-galactosyl sphingosine, increased vacuolization and abnormal endocytic trafficking of lactosylceramide, and over-acidification of the lysosome in patient-derived cells—albeit the pathological mechanism is yet to be understood [7,11].

Most MPSPS patients look healthy in the neonatal period with a normal Apgar score (≥7); however, by the age of two to six months, they begin to experience joint stiffness and recurrent respiratory disturbance with infection. As the disease progresses, other MPS-like symptoms such as hepatosplenomegaly and coarse facial features become apparent. Dysostosis multiplex—a characteristic symptom in MPS—also manifests in MPSPS as vertebral dysplasia (with round and hook-shaped vertebral bodies), widening of anterior ribs, bullet-shaped phalanges, and metacarpal pointing [5]. In our experience, dysostosis multiplex observed by X-ray is evident by the age of one year. In most cases, these characteristic symptoms lead to suspicion of MPS or mucolipidoses (ML), and the MPSPS diagnosis is confirmed via molecular genetic testing. Elevated urinary GAG, detailed patient/family history, and prenatal diagnosis may also contribute to the diagnosis.

Hematopoietic disorders and renal failure are symptoms specific to MPSPS, hence the inclusion of the term “Plus” in the disease name. Our previous study of an autopsy case revealed hypoplastic bone marrow and significant glomerular hyalinization [7]; however, its clinical course was unknown. Furthermore, it was unclear whether growth failure (observed in MPS and ML) was evident in MPSPS. In the present study, we collected and analyzed time-series of all available clinical information—particularly of blood counts, kidney function, and weight/height—to further characterize the clinical phenotype of MPSPS.

## 2. Results

### 2.1. Hematopoietic Impairment in MPSPS

**Figure 1:**
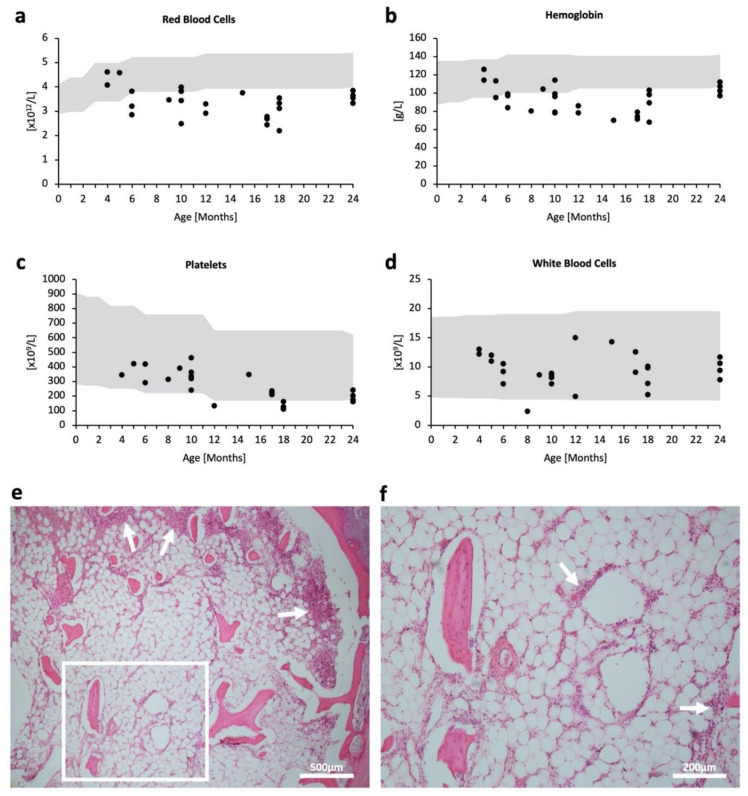
Hematopoietic impairment in MPSPS patients. (**a**–**d**) Scatterplot of respective blood tests in accordance with age (month). Some patients had no data for specific tests; some patients had multiple test data from different time points. Grey areas denote pediatric standard references. (**a**) Red blood cell count (10^12^/L); *n* = 26. (**b**) Hemoglobin level (g/L); *n* = 29. (**c**) Platelet count (10^9^/L); *n* = 24. (**d**) White blood cell count (10^9^/L); *n* = 28. (**e**,**f**) H & E staining of bone marrow from the sternum in an autopsy case. (**e**) White arrows indicate the accumulation of cells. Scale bar 500 μm. (**f**) Magnified image of (**e**) white square. Scale bar 200 μm.

Levels of red blood cells and hemoglobin appeared to decline and deviate from the reference range, starting around six months of age (Figure 1a,b), reflecting progressive anemia. Platelet counts were low within the reference range (Figure 1c). On the other hand, the white blood cell count generally lay within the reference range and seemed to be unaffected by age (Figure 1d). Bone marrow histology showed a markedly hypocellular fatty marrow with the absence of erythroblastic islands and megakaryocytes. Cells—most likely leukocytes such as granulocytes—were present in loose aggregates especially along the trabecular bone, as indicated by white arrows (Figure 1e,f). These results indicate prominent and progressive hematopoietic abnormalities, especially in red blood cells and platelets.

### 2.2. Kidney Impairment in MPSPS

**Figure 2:**
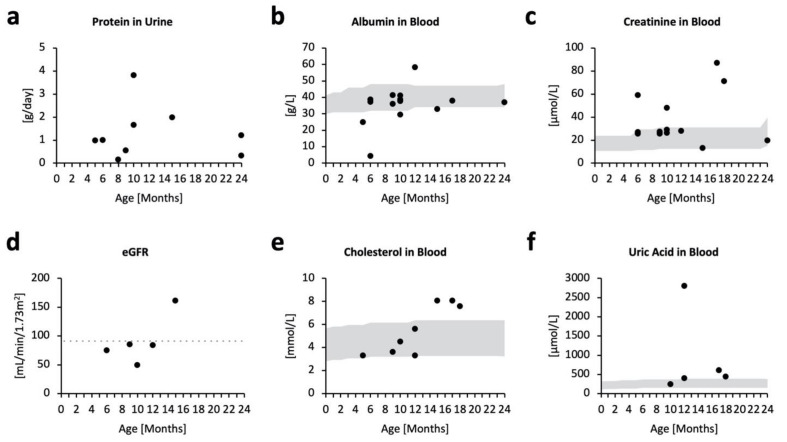
Biochemical markers for kidney function. Scatterplot of respective biochemical test in accordance with age (months). Some patients have no data for specific tests; some patients have multiple test data from different time points. Grey areas denote pediatric standard references. (**a**) Protein in urine (g/day); *n* = 5. (**b**) Albumin in blood (g/L); *n* = 14. (**c**) Creatinine in blood (µmol/L); *n* = 14. (**d**) Estimated glomerular filtration rate (mL/min/1.73 m^2^); *n* = 5. Points below the grey dotted line indicate lower eGFR levels compared with the pediatric reference. (**e**) Cholesterol in blood (mmol/L); *n* = 8. (**f**) Uric acid in blood (µmol/L); *n* = 5.

Proteinuria was evident from as early as five months old, but did not appear to be progressive (Figure 2a). Abnormal excretion of protein in the urine was accompanied by tendencies of blood hypoalbuminemia (Figure 2b) and increased blood creatinine (Figure 2c), suggesting poor kidney function. The estimated glomerular filtration rate (eGFR) was relatively stable over the clinical course. However, four out of five patients had eGFR levels lower than 90, indicating mild kidney damage (Figure 2d). eGFR levels are often overestimated in children <2 years of age, even when pediatric eGFR equations are used, as their low muscle mass is not accounted for. Therefore, kidney impairment in MPSPS patients may be more profound than shown by eGFR levels. Further, cholesterol levels appeared to increase with age (Figure 2e), which may reflect an increased synthesis of albumin to offset their excessive excretion into urine. Most patients experience elevated levels of uric acid in the blood (Figure 2f). These data suggest the occurrence of kidney failure in MPSPS patients.

**Figure 3:**
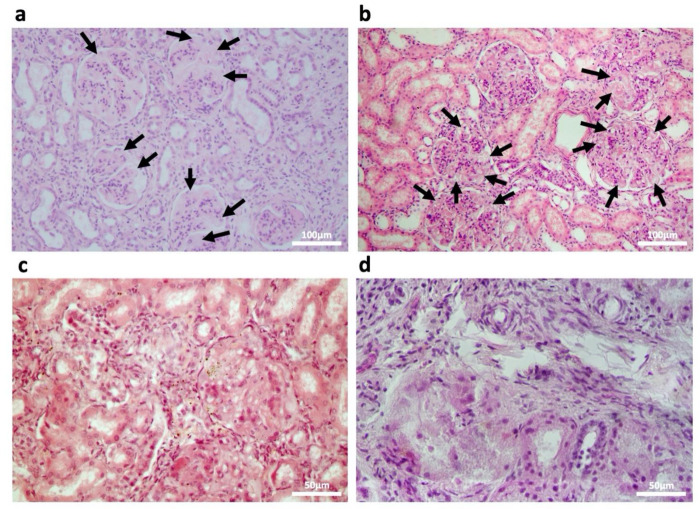
Histology of postmortem kidney tissue from four different MPSPS patients. H&E staining. Foamy podocytes are indicated with black arrows. (**a,b**) Low magnification images; scale bars 100 μm. (**c,d**) High magnification images; scale bars 50 μm.

Histopathological examinations of different MPSPS patients’ kidneys were performed (Figure 3), which confirmed the presence of foamy podocytes characterized by a coarsely vacuolated cytoplasm (Figure 3a,b), chronic interstitial inflammation, and periglomerular fibrosis. Severity differed between patients, and in the most severe case glomerular structures were completely destroyed (Figure 3c,d).

### 2.3. Birth Size and Growth

**Figure 4:**
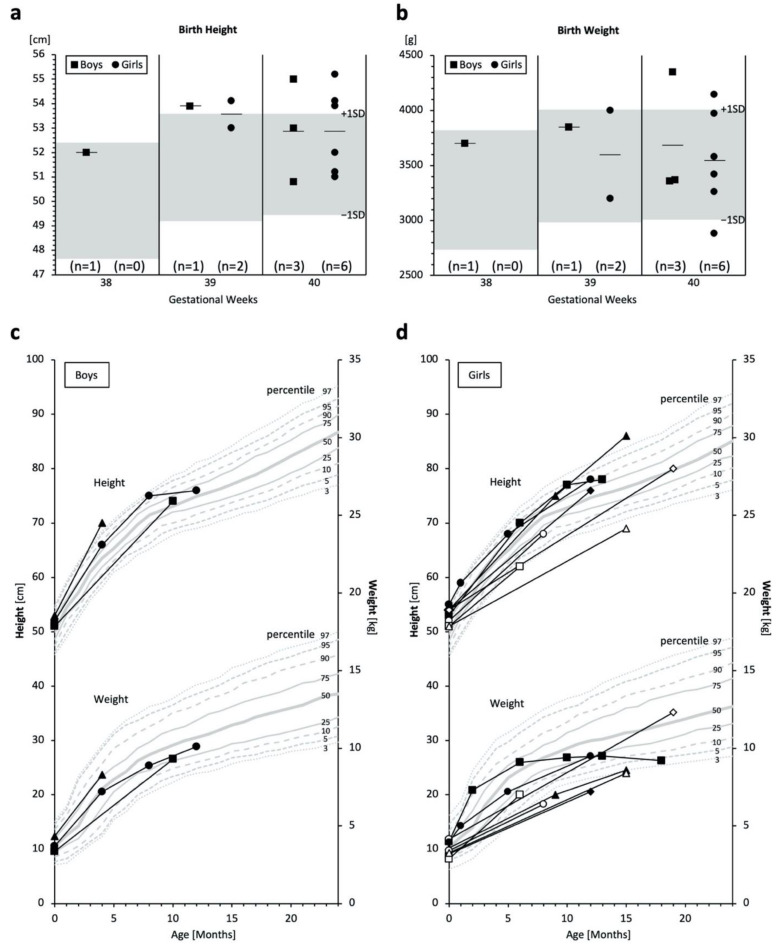
Measurements of birth weights/heights and physical growth. (**a**,**b**) Birth height and weight of MPSPS patients. Boys are indicated by squares and girls by circles. Bars indicate averages. Grey areas denote pediatric standard references ranging between −1 SD and +1 SD of each gestational week. (**c**,**d**) Growth curves; boys *n* = 3; girls *n* = 8. Each unique symbol represents an individual patient. Some patients have only one measurement; some patients have multiple test data from different time points, connected by lines. Grey lines denote the pediatric reference (percentile) of Yakut children.

MPSPS patients in the study were born within the standard gestational period, and most had normal or high birth height and weight measurements (Figure 4a,b). However, in several cases, growth retardation was observed months or one year after birth (Figure 4c,d). Further, MPSPS patients had poor weight gain, and almost all patients showed below-average weight (Figure 4c,d).

## 3. Discussion

Biochemical, blood, and histopathological examinations revealed MPSPS-specific renal and hematopoietic abnormalities (“Plus” symptoms) usually not found in the conventional MPS.

Hematopoietic disorders—including anemia, thrombocytopenia, and coagulopathy with episodic internal bleeding—have been repeatedly described in MPSPS [11]. A recent case report described a Moroccan MPSPS patient [9] with anemia, thrombocytopenia, and normal peripheral white blood cell count, corresponding to our hematopoietic data and bone marrow histology (showing an absence of erythroblastic islands and megakaryocytes). It has also been suggested that splenomegaly, observed in MPSPS patients, contributes to anemia and thrombocytopenia by causing over-filtration and excessive destruction of circulating blood cells. Erythropoietin deficiency caused by renal dysfunction may be contributing to anemia, although erythropoietin has not been measured in any patients.

The pathological mechanism of renal abnormalities in MPSPS is yet to be elucidated, but may be explained in part by similar manifestations in other lysosomal storage diseases (LSDs), specifically Fabry disease (FD) [12]. FD is an X-linked lysosomal storage disorder caused by deficiency of lysosomal enzyme α-galactosidase A (α-Gal A), resulting in accumulation of globotriaosylceramide (Gb3), its deacylated form lyso-Gb3, and related glycolipids. Development of early polyuria, polydipsia, and proteinuria are followed by end-stage renal disease—one of the leading causes of death in male FD patients—during the third to fourth decade of life. Renal histology of FD has been well observed and is characterized by (1) hypertrophic podocytes distended with foamy appearing vacuoles; (2) segmental or global glomerulosclerosis; (3) lipid accumulation in podocyte, parietal epithelial, mesangial, and glomerular endothelial cells; (4) vacuolization in the capillary endothelium and distal tubular epithelial cells, including within Henle’s loop and the collecting duct; (5) deposition in the capillary, arterial, arteriolar endothelial, pericyte, and smooth muscle cells; (6) progressive tubular atrophy; (7) interstitial fibrosis; and (8) varying degrees of glomerular obsolescence [13]. The exact mechanism of renal injury is unclear, but it has been hypothesized that podocytes—which are post-mitotic (irreplaceable when destroyed)—are injured due to the toxic accumulation of Gb3, causing nephrotic syndrome [14].

MPSPS patients exhibit renal dysfunction, albeit with more rapid progression. Of the 18 total MPSPS patients reported thus far, 17 were described having proteinuria before two years of age, six cases of which coincided with nephromegaly and nephrotic syndrome [5,7,8,9,11]. Biochemical analyses presented in this study similarly indicate albuminemia, proteinuria, and elevated creatinine, cholesterol, and uric acid. Proteinuria is very apparent (more than 1 g per day) and is presumed to have appeared early, although the earliest data is from five months of age. Histopathological examinations of patient kidney sections reveal a notable presence of foamy podocytes, as well as chronic interstitial inflammation and periglomerular fibrosis. In particular, foamy podocytes seen in MPSPS were similar to those seen in Fabry disease [15,16,17] or mucolipidosis III [18], with numerous lipid inclusion bodies apparently causing mild mesangial widening. The histological resemblance between MPSPS and FD renal manifestations may be explained by the accumulation of sphingolipids (cholesterol/lactosylceramide and Gb3/lyso-Gb3/glycolipids, respectively) in both diseases [11,14], as it has been reported that inflammatory responses caused by metabolic aberrations lead to foam cell formation. Further studies are clearly needed to uncover the pathophysiology of renal complications in MPSPS.

Data for the physical growth of MPSPS patients are limited due to non-frequent measurements in public health care systems or pediatrics. From the limited data, birth height and weight appear to be within or higher than the normal range. However, growth disturbances became evident in the clinical course of the disease, and many of the subjects showed lower height and weight than the age-matched average after six months or one year old. Due to the low life expectancy of MPSPS patients and limited data, we are unable to conclude whether short stature or growth failure are key features of MPSPS.

This is a retrospective study and the data are limited. It has been speculated that rapidly evolving cardiopulmonary failure contributes to short life expectancy; however, our limited data did not allow for formal analysis regarding the precise cause of death in MPSPS. There are no specific treatments available for MPSPS patients, although supportive or symptomatic management may temporarily improve the quality of life for patients and their families [5]. It has been reported that a Moroccan MPSPS patient, whose hemophagocytic lymphohistiocytosis was successfully treated with steroids, has survived to his sixth year of age (significantly improved life expectancy compared to MPSPS patients in Yakutia, Russia)—demonstrating that if patients overcome infection and inflammation in early childhood, phenotypes may stabilize thereafter [9]. Accumulation of clinical data and therapeutic trials, as well as basic science research on the function of mutated VPS33A, are expected to elucidate the pathomechanisms of MPSPS.

## 4. Materials and Methods

### 4.1. Clinical Cases

Clinical data were collected from medical records of MPSPS patients who died from 2007 to 2019 in the Republic Hospital No.1—National Center of Medicine (Yakutsk, Russia). Diagnoses of all patients were confirmed via genetic testing at the Republic Hospital No.1—National Center of Medicine. Autopsies were carried out for five patients. All clinical and biochemical data from medical records and histological images were retrospectively reviewed.

### 4.2. Histological Analyses

Autopsy was performed on five patients, and tissue samples were obtained. The following fixation procedure was used: (1) specimens were placed in the fixative solution immediately after collection, with a 20:1 volume ratio between fixing agent and tissue sample, (2) a portion of each sample was fixed in 10% neutral buffered formalin (NBF) for 24 h, processed into paraffin blocks, sectioned, and stained with routine hematoxylin and eosin. Sections had a thickness of no more than 5 mm and side dimensions of about 1 cm.

### 4.3. Blood Count, Biochemical, and Physical Standard References

Japanese pediatric references [19] were used for hematopoietic and biochemical analyses, because standard pediatric references for Yakut children were not available. Estimated glomerular filtration rate (eGFR) was calculated using the Japanese Pediatric eGFR equation $\left[0.349\times height\;\left(cm\right)÷Serum\;Creatinine\;\left(\frac{mg}{dL}\right)\right]$ [20]. Yakut references [21] were used for birth height and birth weight. Russian references were used for growth curves [22].

## Data Availability

All relevant data, which supports the findings of the study, are within the manuscript.
